# Substance Use Disorders and Adoption: Findings from a National Sample

**DOI:** 10.1371/journal.pone.0049655

**Published:** 2012-11-15

**Authors:** Gihyun Yoon, Joseph Westermeyer, Marion Warwick, Michael A. Kuskowski

**Affiliations:** Minneapolis VA Health Care System, University of Minnesota Medical School, Minneapolis, Minnesota, United States of America; University of Granada, Spain

## Abstract

**Background:**

Prior research has shown that adoptees have a higher rate of substance use disorders (SUDs) than nonadoptees. But these findings have not been verified with a population-based sample of adult adoptees in the United States. Also, no previous adoption study has measured the prevalence of each specific substance use disorder (SUD). We aimed to compare lifetime prevalence rates and odds ratios of SUDs in adopted and nonadopted adults.

**Methods and Findings:**

The data come from the National Epidemiologic Survey on Alcohol and Related Conditions (NESARC). The main outcome measure was the prevalence of lifetime SUDs in adopted (n = 378) and nonadopted adults (n = 42503). Adoptees and nonadoptees were compared to estimate the odds of lifetime SUDs using logistic regression analysis. Adoptees had higher prevalence rates of lifetime SUDs than nonadoptees. Overall, adoptees had a 1.87-fold increase (adjusted odds ratio [AOR] 1.87, 95% CI 1.51–2.31) in the odds of any lifetime SUD compared to nonadoptees. For each SUD, adoptees had higher odds for alcohol abuse/dependence (AOR 1.84), nicotine dependence (AOR 1.78), cannabis abuse/dependence (AOR 1.77), cocaine abuse/dependence (AOR 2.54), amphetamine abuse/dependence (AOR 3.14), hallucinogen abuse/dependence (AOR 2.85), opioid abuse/dependence (AOR 2.21), and other drug abuse/dependence (AOR 2.87) compared to nonadoptees. This study also identified two adoption-specific risk factors (Hispanic, never married) associated with any lifetime SUD.

**Conclusions:**

This study demonstrated an increased risk of lifetime SUDs in adopted adults. The findings can be useful for clinicians and policy makers to provide education, prevention, and support for adoptees and their families.

## Introduction

The annual number of adopted children in the United States increased 15% from 118,779 in 1990 to 136,000 in 2008 [1]. There are many positive aspects of adoption. The large majority of adopted children are well adjusted [2], with mental health profiles similar to nonadopted children [3]. Placement in an adoptive family most likely results in better childhood experiences, health care, family stability, and family relationships [4]. Adoption has been associated with increased cognitive development and cognitive competence among adopted children [5].

Nevertheless, compared to nonadopted persons, adoptees still have higher rates of psychiatric disorders [6]–[12] including substance use disorders (SUDs). Recently two large European population-based studies reported that intercountry adoptees have an increased risk of SUDs [6], [7]. A Swedish study using a national cohort showed higher odds for drug abuse (odds ratio: 5.2) and alcohol abuse (odds ratio: 2.6) among intercountry adoptees as compared to nonadoptees [7]. In a study of young adult intercountry adoptees comparing with nonadoptees in the Netherlands, adoptees were 2.05 times more likely to have substance abuse or dependence [6].

Several studies in the United States also reported higher rates of SUDs among adoptees. We previously reported that the proportion of adoptees was 14 times higher than expected in two SUD treatment programs [13]. In a national US school survey, adopted adolescents had higher smoking, drinking, and drunk scores than nonadopted adolescents [14]. But these rates have not yet been investigated in a representative sample of US adult adoptees.

The higher SUD rates among adoptees are associated with genetic and environmental risk factors [15]. Genetic risk factors for SUDs include alcoholic biological parents [16], [17], psychiatric disorders in biological parents [17], and male gender [18]. These vulnerabilities in adoptees can be exacerbated by environmental risk factors for SUDs such as alcoholic adoptive parents [19], three or more pre-adoption placements [20], fetal alcohol effects [21], psychological factors unique to adopted children [22], and higher scores on the adverse adoptive environment scale (consisting of substance use, psychiatric conditions, and legal problems in adoptive parents) [18]. This is important information, but there is a need to study risk factors associated with SUD specifically for adopted persons.

The purpose of this study was to investigate lifetime prevalence rates and odds ratios of SUDs comparing adoptees and nonadoptees in a nationally representative sample in the United States. We hypothesized that lifetime prevalence rates of SUDs are higher in adoptees than nonadoptees. We also investigated which demographic variables are risk factors for SUD specific to adoption. To our knowledge, this is the first study investigating odds ratios for SUDs among adopted and nonadopted adults using a large population-based sample in the United States.

**Table 1:** Demographic characteristics of adopted and nonadopted individuals.

	Adopted	Nonadopted	
Characteristics	(n = 378)	(n = 42503)	OR (95% CI)
**Gender**			
Male	158 (41.8%)	18258 (43.0%)	1 (Reference)
Female	220 (58.2%)	24245 (57.0%)	1.05 (0.85–1.29)
**Age (years)**			
18–29	78 (20.6%)	8544 (20.1%)	1.30 (0.99–1.71)
30–44	154 (40.7%)	13154 (30.9%)	**1.67 (1.33–2.09)**
45 or older	146 (38.6%)	20805 (48.9%)	1 (Reference)
**Race/Ethnicity**			
White	253 (66.9%)	24133 (56.8%)	1 (Reference)
Hispanic	42 (11.1%)	8236 (19.4%)	**0.49 (0.35–0.68)**
Black	48 (12.7%)	7975 (18.8%)	**0.57 (0.42–0.78)**
Native American	10 (2.6%)	819 (1.9%)	1.17 (0.62–2.20)
Asian/Pacific Islander	25 (6.6%)	1340 (3.2%)	**1.78 (1.18–2.69)**
**Education (years)**			
0–11	49 (13.0%)	7763 (18.3%)	1 (Reference)
12	92 (24.3%)	12388 (29.1%)	1.18 (0.83–1.67)
13–15	138 (36.5%)	12470 (29.3%)	**1.75 (1.26–2.43)**
16 or more	99 (26.2%)	9882 (23.3%)	**1.59 (1.13–2.24)**
**Personal income ($)**			
0–19999	167 (44.2%)	20836 (49.0%)	1 (Reference)
20000–34999	90 (23.8%)	9832 (23.1%)	1.14 (0.88–1.48)
35000–59999	76 (20.1%)	7587 (17.9%)	1.25 (0.95–1.64)
60000 or more	45 (11.9%)	4248 (10.0%)	1.32 (0.95–1.84)
**Marital status**			
Married/cohabitation	201 (53.2%)	21834 (51.4%)	1 (Reference)
Divorced/separated	63 (16.7%)	6778 (15.9%)	1.01 (0.76–1.34)
Never married	90 (23.8%)	9657 (22.7%)	1.01 (0.79–1.30)
Widowed	24 (6.3%)	4234 (10.0%)	**0.62 (0.40–0.94)**

Abbreviations: OR, odds ratio; CI, confidence interval.

**Table 2:** Prevalence rates and adjusted odds ratios of lifetime SUDs in adopted and nonadopted individuals.

	Adopted	Nonadopted		
	(n = 378)	(n = 42503)		
Disorder	Prevalence (%)	Prevalence (%)	OR (95% CI)	AOR^a^ (95% CI)
**Any SUD**	50.5	35.4	1.87 (1.53–2.29)	**1.87 (1.51–2.31)**
**Licit SUD**	49.2	34.1	1.87 (1.53–2.29)	**1.88 (1.52–2.32)**
Alcohol abuse/dependence	41.0	27.5	1.83 (1.49–2.25)	**1.84 (1.48–2.29)**
Alcohol abuse	23.5	17.8	1.42 (1.12–1.81)	**1.39 (1.08–1.77)**
Alcohol dependence	19.0	11.1	1.89 (1.46–2.45)	**1.83 (1.40–2.39)**
Nicotine dependence	25.4	16.1	1.78 (1.41–2.24)	**1.78 (1.41–2.25)**
**Illicit SUD**	16.9	9.4	1.96 (1.50–2.58)	**1.89 (1.43–2.50)**
Any illicit drug abuse	14.8	8.2	1.94 (1.46–2.58)	**1.86 (1.39–2.50)**
Any illicit drug dependence	5.0	2.4	2.14 (1.35–3.41)	**2.08 (1.30–3.32)**
Cannabis abuse/dependence	13.2	7.6	1.85 (1.37–2.49)	**1.77 (1.30–2.40)**
Cocaine abuse/dependence	6.6	2.7	2.59 (1.72–3.90)	**2.54 (1.68–3.83)**
Amphetamine abuse/dependence	5.6	1.7	3.31 (2.12–5.17)	**3.14 (2.00–4.92)**
Hallucinogen abuse/dependence	4.2	1.4	3.06 (1.84–5.08)	**2.85 (1.70–4.76)**
Opioid abuse/dependence	2.9	1.3	2.22 (1.21–4.06)	**2.21 (1.20–4.05)**
Other drug abuse/dependence^b^	4.0	1.4	2.95 (1.75–4.98)	**2.87 (1.70–4.87)**

Abbreviations: SUD, substance use disorder; OR, odds ratio; AOR, adjusted odds ratio; CI, confidence interval.

a Adjusted for gender, age, race, education, and marital status.

b Other drug includes sedatives, tranquilizers, inhalants, solvents, and others.

## Methods

This study was approved by the Research & Development Committee of Minneapolis VA Health Care System. Oversight by the Institutional Review Board was exempted because the current study used de-identified public data.

### Sample

The study data were drawn from the National Epidemiologic Survey on Alcohol and Related Conditions (NESARC) [23], [24]. The NESARC is a nationally representative survey of 43093 adults in the United States. The nationwide face-to-face survey was conducted in 2001 and 2002 by the US Census Bureau and the National institute on Alcohol Abuse and Alcoholism. The overall response rate for the 2001–2002 NESARC was 81 percent. The survey provides data on alcohol and drug use, psychiatric classification of SUDs and other psychiatric disorders, treatment utilization, and sociodemographic information. The NESARC procedures were reviewed and approved by the US Census Bureau and the US Office of Management and Budget. Informed consent was obtained from all NESARC participants.

### Measures

#### Adoption status

Adoption status was determined by face-to-face structured interviews with the following two questions: (1) “Did you live with at least one of your biological or birth parents at any time when you were growing up, that is before you were 18 years old?” When participants answered “no,” the following questions were asked: (2) “When you were growing up, before the age of 18, were you raised by adoptive parents?” In this study, adoptees were defined when the question #1 was answered “no” and the question #2 was answered “yes.” By this criteria, adopted individuals in this study were raised by adoptive parents, but never lived with any biological parents when they were growing up.

#### Lifetime substance use disorders

Diagnoses of lifetime SUDs were made by the Alcohol Use Disorder and Associated Disabilities Interview Schedule–DSM-IV Version (AUDADIS-IV) [25], which was developed based on the Diagnostic and Statistical Manual of Mental Disorders, Fourth Edition (DSM-IV) [26]. The AUDADIS-IV has shown to be reliable and valid for assessing SUDs [27]–[30]. In a general population sample testing the AUDADIS-IV, the reliabilities were good to excellent for DSM-IV lifetime alcohol abuse and dependence (kappa = 0.70), lifetime nicotine dependence (kappa = 0.60), any drug abuse and dependence (kappa = 0.66–0.79), cannabis abuse and dependence (kappa = 0.71–0.78), cocaine abuse and dependence (kappa = 0.68–0.91), and heroin abuse and dependence (kappa = 0.66–0.80) [28], [29].

Lifetime SUDs evaluated were as follows: (1) nicotine dependence; (2) alcohol abuse or dependence; (3) cannabis abuse or dependence; (4) cocaine abuse or dependence; (5) opioid (painkillers or heroin) abuse or dependence; (6) amphetamine abuse or dependence; (7) hallucinogen abuse or dependence; and (8) other drug (sedatives, tranquilizers, inhalants, solvents, or others) abuse or dependence. Although sedatives, tranquilizers, inhalants, and solvents were independently assessed, we have lumped into the category of “other drug” because of the small percentages of these drug disorders.

**Table 3:** Logistic regression of adoption-specific risk factors associated with any lifetime SUD in all individuals (n = 42881).

	Any lifetime SUD	
Adoption-specific risk factor	OR	95% CI
**Gender**		
Male	1	Reference
Female	0.95	0.62–1.45
**Age (years)**		
18–29	0.82	0.47–1.42
30–44	0.69	0.39–1.20
45 or older	1	Reference
**Race/Ethnicity**		
White	1	Reference
Hispanic	**2.52**	**1.30–4.89**
Black	1.20	0.64–2.25
Native American	0.97	0.27–3.55
Asian/Pacific Islander	1.39	0.57–3.37
**Education (years)**		
0–11	1	Reference
12	1.10	0.55–2.22
13–15	0.74	0.38–1.43
16 or more	1.40	0.70–2.80
**Marital status**		
Married/cohabitation	1	Reference
Divorced/separated	0.91	0.51–1.60
Never married	**1.71**	**1.02–2.84**
Widowed	1.23	0.49–3.10

Abbreviations: SUD, substance use disorder; OR, odds ratio; CI, confidence interval.

### Statistical Analyses

Participants were classified into two groups based on adoption status (adopted and nonadopted). Prevalence rates of demographic variables and lifetime SUDs were obtained using cross-tabulations. Comparison of the two groups was performed for demographic variables to estimate odds ratios (OR) and 95% confidence intervals using a series of logistic regression. The variables (1) age, (2) race, (3) education, (4) personal income, and (5) marital status were coded as indicator variables using (1) ages of 45 or older, (2) white, (3) education of 0–11 years, (4) personal income of $0–19999, and (5) married/cohabitation as the reference category.

For main outcome variables (prevalence rates of lifetime SUDs), we conducted logistic regression to examine the effects of adoption status on lifetime SUDs. First, unadjusted odds ratios (OR) and 95% confidence intervals were estimated. Second, adjusted odds ratios (AOR) and 95% confidence intervals were estimated while adjusting for covariates (gender, age, race, education, and marital status). The covariates were selected based on the known correlates of adoption or of SUD.

To identify adoption-specific risk factors for any lifetime SUD, a series of logistic regression analyses were conducted using the entire sample. Interaction effects between adoption status and other predictor variables (gender, age, race, education, and marital status) were examined in logistic models to determine if any of these variables modified the effect of adoption status on SUD.

## Results

### Demographic Characteristics

Of the 43093 adults in the NESARC, 42881 participants (99.5%) were included for data analysis after excluding 212 individuals who did not know their adoption status. The rate of adoptees (n = 378) in this study sample (n = 42881) was 0.88%.

Table 1 describes the demographic characteristics of two groups based on the adoption status. Adoptees were more likely than nonadoptees to be younger (ages of 30–44), Asian/Pacific Islanders, and to have higher levels of education. Adoptees were less likely than nonadoptees to be Hispanic, black, or widowed. The remaining demographic characteristics (gender, personal income) failed to show statistical differences.

### Prevalence and Odds Ratios of Substance Use Disorders

Table 2 presents prevalence rates of lifetime SUDs in the two adoption groups. Prevalence of any lifetime SUD was 43% higher among adoptees (50.5%) compared to nonadoptees (35.4%). Adoptees had higher prevalence rates for every SUD compared to nonadoptees. Lifetime prevalence rates of licit SUDs were 41.0% (alcohol) and 25.4% (nicotine) among adoptees. In nonadoptees, the rates dropped to 27.5% (alcohol) and 16.1% (nicotine). Lifetime prevalence rates of illicit SUDs ranged from 2.9% (opioid) to 13.2% (cannabis) among adoptees and from 1.3% (opioid) to 7.6% (cannabis) among nonadoptees.

Table 2 also shows unadjusted odds ratios (OR), adjusted odds ratios (AOR), and 95% confidence intervals (95% CI). Logistic regression analyses showed that the odds of every lifetime SUD were statistically higher among adoptees than nonadoptees after adjusting for confounding variables. Overall, adoptees had a 1.87-fold increased risk of any lifetime SUD than nonadoptees (AOR 1.87, 95% CI 1.51–2.31). For licit SUD, adoptees had a 1.88-fold increased risk than nonadoptees (AOR 1.88, 95% CI 1.52–2.32). For illicit SUD, adoptees had a 1.89-fold increased risk than nonadoptees (AOR 1.89, 95% CI 1.43–2.50). The adjusted odds ratios of SUDs ranged from 1.77 (cannabis) to 3.14 (amphetamine).

Adoptees also had higher odds of both diagnostic categories of abuse and dependence compared to nonadoptees as follows: alcohol abuse (AOR 1.39, 95% CI 1.08–1.77), alcohol dependence (AOR 1.83, 95% CI 1.40–2.39), any illicit drug abuse (AOR 1.86, 95% CI 1.39–2.50), and any illicit drug dependence (AOR 2.08, 95% CI 1.30–3.32).

### Adoption-Specific Risk Factors for Any Substance Use Disorders

Table 3 presents adoption-specific risk factors for any lifetime SUD among all adopted and nonadopted individuals (n = 42881). Logistic regression examined interaction effects between adoption status and risk factor variables (gender, age, race, education, and marital status). Two adoption-specific risk factors were significantly associated with any lifetime SUD: (1) Hispanic (OR 2.52, 95% CI 1.30–4.89) and (2) never married (OR 1.71, 95% CI 1.02–2.84). The strongest adoption-specific risk factor was Hispanic race-ethnicity, which was 2.52 times more likely associated with any lifetime SUD. The second adoption-specific risk factor was being never married, which increased the odds of any lifetime SUD by a factor of 1.71.

## Discussion

### Odds Ratios for Substance Use Disorders

Our findings from the population-based NESARC data showed that adoptees have higher rates of lifetime SUDs than nonadoptees. These findings are consistent with two large European studies [6], [7], although the European data were based on intercountry adoptees and our data were based on predominantly domestic adoptees. The American NESARC rate of any lifetime SUD among adoptees (AOR 1.87, 95% CI 1.51–2.31) overlap with the rates of alcohol abuse (AOR 2.6, 95% CI 2.0–3.3) among Swedish intercountry adoptees [7] and the rate of substance abuse or dependence (AOR 2.05, 95% CI 1.32–3.17) among a cohort of intercountry adoptees in the Netherlands [6]. These comparisons among three countries indicate that the increased prevalence for SUD tends to hold true regardless of country of adoption (Sweden, Netherlands, United States) and mode of sampling adoptees (intercountry vs. all adoptees; cohort vs. cross section of all adoptees in one time period). One possible discrepancy could be drug abuse. The overall rate of illicit SUD in the NESARC data (AOR 1.89, 95% CI 1.43–2.50) did not overlap with the Swedish drug abuse rate (AOR 5.2, 95% CI 2.9–9.3). Concurrently, the NESARC rates for cocaine, amphetamine, hallucinogen, opioid, other drug abuse/dependence, and any illicit drug dependence did overlap with Swedish drug abuse rate, suggesting that type of drug, type of adoptee, and/or sampling method could produce even higher odds ratios for SUD. In particular, intercountry adoptees in the European studies could have higher odds of mental health problems than domestic adoptees in the NESARC study because of complicated intercountry adoption process, acculturation, and racial issues [31], as well as delays in adoption so that children are older. Although some disagreement in specific rates suggests national/cultural or methodological differences, these differences are more one of extent than of general directionality.

Importantly, adoptees’ odds ratios were high for both abuse and dependence (not just dependence alone). The odds ratios were slightly higher for dependence than abuse, but these differences were not significant on the 95% CI. This findings applied to both alcohol abuse/dependence as well as drug abuse/dependence. From a public health perspective, these data indicate that adoptees have a wide range of SUD including abuse and dependence.

These adoption studies convey both theoretical and practical implications. On a theoretical level, they indicate a linkage between being adopted and having a propensity to SUD years later in adulthood. The most likely linkage is a genetic one [32]–[35] with parental SUD genes (or perhaps related genes such as impulsivity [36]) favoring (1) a genetic propensity for SUD in their offspring and/or (2) selection of more severe parental SUD due to their inability to raise their children. Other biological factors include paternal fetal damage from impaired spermatogenesis, detrimental maternal intrauterine or childbirth events [21], and – in those adopted later – childhood exposure to drug use and adverse child raising [37]. On the contrary, adopted children tend to have adoptive families with social characteristics better than families at large, including higher education and higher socioeconomic status. In sum, adopted children tend to have a unique admixture of greater at-risk genetic factors and enhanced environmental factors in the adoptive family [17], [38]. Whatever the cause, being adopted should be recognized as a risk factor to SUD.

### Adoption-Specific Risk Factors for Any Substance Use Disorders

Our study identified two risk factors increasing the prevalence of lifetime SUD specific to adoption status. The first and largest risk factor was Hispanic race-ethnicity (AOR 2.52, 95% CI 1.30–4.89). Factors in the Hispanic adoptees that might account for this greater vulnerability to SUD include a higher prevalence of foreign-born adoptees with more adverse intrauterine factors (poor nutrition, impoverished family-of-origin) and/or environmental factors in the family-of-adoption (e.g., adopted by Hispanic relatives with lower socioeconomic status as opposed to the usual families-of-adoption with higher socioeconomic status). Or conversely, Hispanic nonadoptees may possess protective factors against SUD, such as higher levels of parental monitoring against SUD [39].

The second risk factor was single marital status (AOR 1.71, 95% CI 1.02–2.84). However, we have no previous studies pointing to a higher single marital status as observed in this study. Other remaining demographic characteristics (gender, age, education) failed to show statistical differences. Although male gender was a risk factor for SUD in population studies [40], [41], it was not an “adoption-specific” risk factor for SUD in this study.

### Limitations

Several limitations should be considered in interpreting our findings. First, the NESARC data did not provide information regarding the status of international or domestic adoption [10], [42]. We assumed it would be similar to percentages of international (15%) and domestic (85%) adoptees estimated previously in the United States [43]. Second, the NESARC also did not provide information on the age at adoption [3] or resilient factors such as self-esteem [44], which could play intermediary roles between adoptees and later SUD. Third, the NESARC did not collect information on SUD and other behaviors in adoptees’ biological parents. Fourth, the sample size of adoptees was disproportionately smaller than that of nonadoptees.

### Conclusions

Adoptees had higher odds for lifetime SUDs than nonadoptees in this study using NESARC data. Despite the advantages of adoptees’ higher educational levels probably due to being raised by higher educated, higher income adopting parents, adoptees are still at higher risk to lifetime SUD. Awareness of adopted persons and their adoptive parents to this risk may help in primary prevention (never using substances; having conservative rules about doses and frequency of use) and in secondary prevention (being alert to early signs and symptoms; timely intervention to reduce damage and increase the chance of recovery). The findings can also be useful for clinicians and policy makers to provide education, prevention, and support for adoptees and their families.
